# Causal relationship between primary sclerosing cholangitis and systemic lupus erythematosus: a bidirectional Mendelian randomization study

**DOI:** 10.1186/s40001-024-01941-1

**Published:** 2024-06-28

**Authors:** Ziwen Pan, Weijie Zhang

**Affiliations:** Institute of Organ Transplantation, Tongji Hospital, Tongji Medical College, Huazhong University of Science and Technology; Key Laboratory of Organ Transplantation, Ministry of Education; NHC Key Laboratory of Organ Transplantation; Key Laboratory of Organ Transplantation, Chinese Academy of Medical Sciences, Wuhan, 430030 China

**Keywords:** Primary sclerosing cholangitis, Systemic lupus erythematosus, Mendelian randomization

## Abstract

**Background:**

Observational studies have found a link between two autoimmune diseases, namely, primary sclerosing cholangitis (PSC) and systemic lupus erythematosus (SLE). However, the relationship remains unclear.

**Methods:**

Bidirectional Mendelian randomization (MR) analysis and statistical methods, including inverse variance weighting, weighted median, and MR-Egger tests, were performed using data from genome-wide association studies to detect a causal relationship between PSC and SLE. Sensitivity analyses were subsequently performed to assess the robustness of the results. Univariate MR methods were also investigated.

**Results:**

Results of MR analysis suggested that PSC was associated with an increased risk for SLE (odds ratio: 1.33, 95% confidence interval: 1.10–1.61, P=0.0039) However, SLE had no significant causal relationship with PSC.

**Conclusion:**

Results of MR analysis revealed that patients with PSC were at an increased risk for SLE, which provides new insights into the relationship between these two autoimmune diseases.

**Supplementary Information:**

The online version contains supplementary material available at 10.1186/s40001-024-01941-1.

## Introduction

Primary sclerosing cholangitis (PSC) is an autoimmune disease of unknown etiology that causes intrahepatic and extrahepatic cholangitis and fibrosis, leading to bile duct stenosis, biliary cirrhosis, and liver failure. The clinical manifestations of PSC are diverse and its diagnosis relies mainly on cholangiography and liver biopsy. The incidence of PSC in Europe and North America is approximately 0.62 and 0.53/100,000 per-person years, respectively [1]. The prevalence of PSC ranges from 0 to 31.7 per 100,000 individuals [1]. Despite its rarity, PSC is the fifth most common indication for liver transplantation in the United States [2].

The causes of PSC are relatively complex, and possible mechanisms include genetic [3] and environmental factors [4]. Because the cause of PSC is currently unclear, there is no specific treatment strategy, and liver transplantation is the only effective treatment. Nevertheless, these patients remain susceptible to recurrence after liver transplantation, which leaves them vulnerable to heavy economic burden. Studies [5] have found that autoantibodies are common in PSC and can be associated with autoimmune disease(s), of which approximately 1.7% are associated with systemic lupus erythematosus (SLE) [6].

SLE is an autoimmune disease characterized by an immune system that attacks healthy cells and tissues throughout the body. A previous literature report [7] suggested that the incidence rate of SLE in North America is 29.1 per 100,000 individuals, which is the highest worldwide. Many studies have investigated the correlation between PSC and autoimmune disease(s) (including inflammatory bowel disease [IBD]) [8]; however, its association with SLE remains unclear. Kadokawa et al. [9]searched the literature and found that 3 case reports related to PSC and SLE were published before 2003, and there is no clear evidence that these two diseases are related. Missoum et al. [10]counted the autoantibody profiles of 3182 Moroccans with autoimmune diseases and found that antinuclear antibodies (ANA) were present in 63% of SLE patients and 50% of PSC patients. However, the etiopathological association between these two autoimmune diseases is still unclear. Therefore, clarifying the connection between these two autoimmune diseases is extremely important for understanding disease pathogenesis and updating specific treatment strategies.

Traditional research methods, such as observational and retrospective studies, measure statistically significant associations between exposure and outcomes; however, these methods make it difficult to draw definitive causal conclusions. Relevant confounders have been identified, measured, and appropriately adjusted because associations could not be determined [11]. Mendelian randomization (MR) [12] primarily uses genetic tools to assess the causal relationship between exposure and outcome; as such, it has advantages over traditional research methods. Therefore, this study used MR to analyze data from 14,627 patients with SLE and 14,890 with PSC to explore the causal relationship between these 2 autoimmune diseases.

## Methods

### Study design

**Fig. 1:**
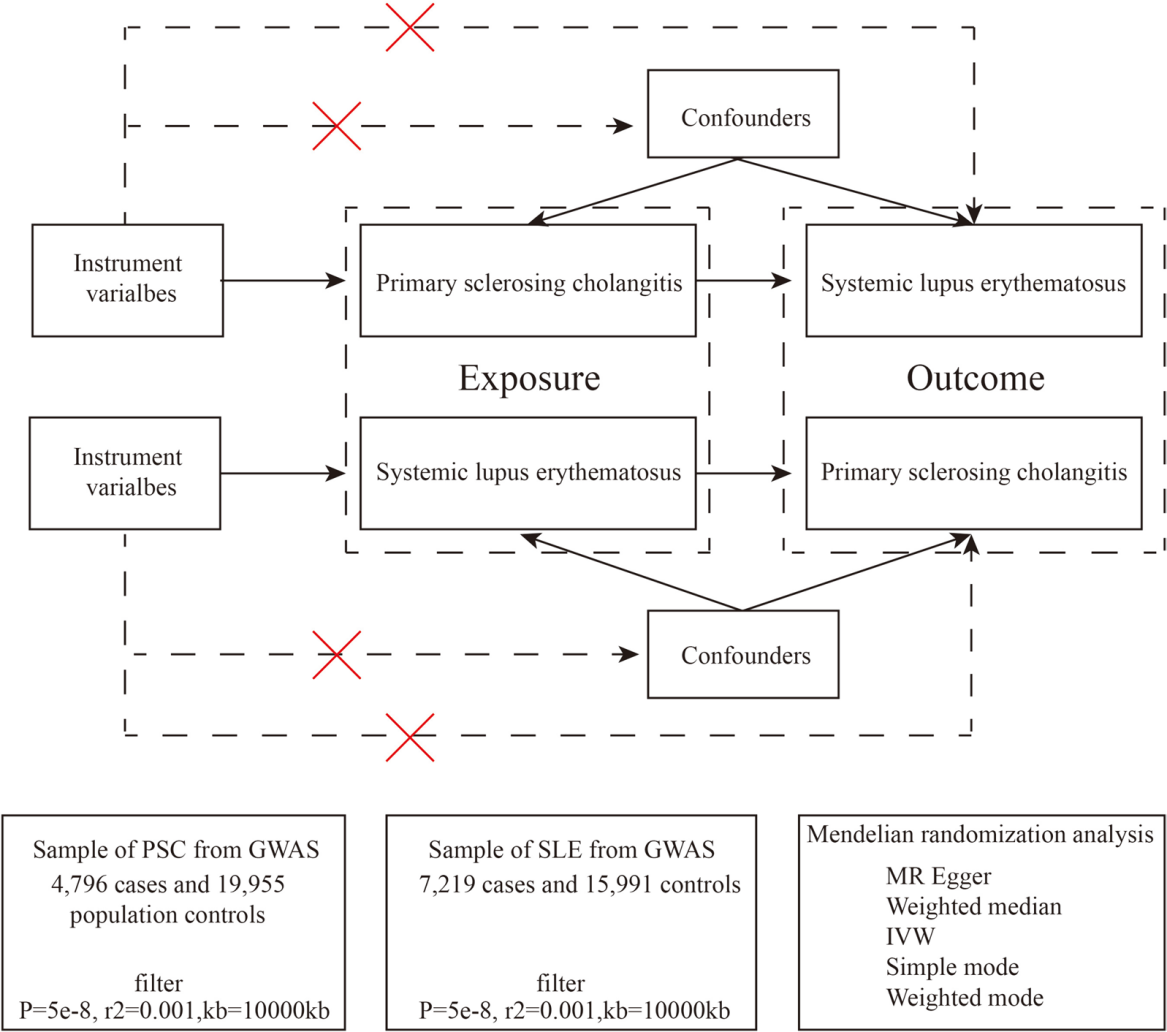
Flow-diagram illustrating the study design

MR analysis is based on three assumptions [13]: (1) Instrumental variables are strongly related to exposure factors; (2) The instrumental variable is not related to any confounders between exposure and outcome; (3) Instrumental variables can only affect outcomes through exposure. A two-way MR method was used to analyze the causal relationship between PSC and SLE. A flow-diagram is presented in Fig. 1.

### Data source

PSC- and SLE-related single-nucleotide polymorphisms (SNPs) were sourced from 24,751 [14] and 23,210 [15] individuals of European ancestry, respectively, in the genome-wide association studies (GWAS) database (Table 1). Patient consents were obtained by corresponding studies.

**Table 1:** GWAS datasets for SLE and PSC

Disease	Study	Journal	Cases	Controls	Sample size	SNP	Datasets in the GWAS	PMID
SLE	Bentham J et al	Nat. Genet	5201	9066	14,267	45	ebi-a-GCST003156	26502338
PSC	Ji et al	Nat. Genet	2871	12,019	14,890	18	ieu-a-1112	27992413

### SNP selection

Strongly (*P* < 5 × 10^–8^) and independently (kb=10000 and r2 < 0.001) screened out SNPs, harmonizing the exposure and outcome data. The threshold was set at *P* < 5 × 10^–8^ using the PhenoScanner database (http://www.phenoscanner.medschl.cam.ac.uk) to eliminate instrumental variables (IVs) that could produce multiple effects (i.e., IBD is a risk factor for PSC [8]).

### Statistical analysis

Five methods (MR-Egger, weighted median, inverse variance weighted [IVW], simple mode, and weighted mode) were used to analyze the causal relationship between the two diseases. IVW is a method that combines the Wald ratio of each SNP to obtain a summary causal estimate [16]. MR-Egger is a method that correlates all SNP results without being affected by pleiotropy [17]. The weighted median method is a method that can accurately calculate causality even when less than 50% of genetic variations are invalid instrumental variables [16, 18]. Simple mode can provide robustness to pleiotropic effects [19, 20]. Weighted modes are sensitive to bandwidth selection difficulties in mode estimation [20]. IVW is used as the main analysis method, and the other four methods are used as auxiliary analysis methods. MR-Egger and IVW were the main methods used for heterogeneity analysis. Heterogeneity was standardized using Cochran’s Q test. MR-Egger regression was used to test for pleiotropy. A “leave-one-out” sensitivity analysis was performed. Finally, a reverse MR analysis of PSCs and SLE was performed. MR results are expressed as odds ratio (OR) and corresponding 95% confidence interval (CI). R version 4.3.1 and the “TwoSampleMR” package, version 0.5.7 (R Foundation for Statistical Computing, Vienna, Austria), were used for data analysis and visualization.

## Results

### Impact of PSC on SLE

**Fig. 2:**
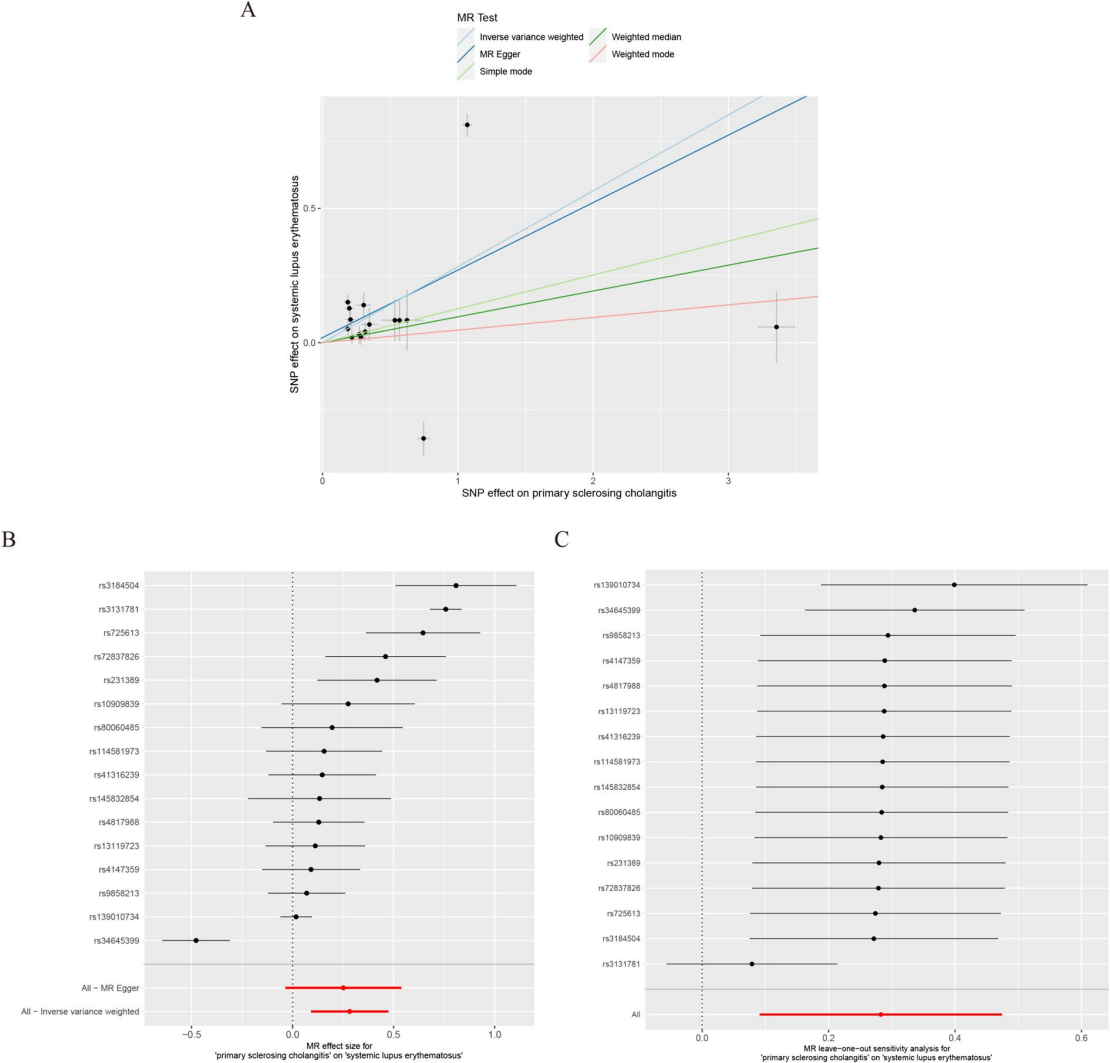
Mendelian randomization estimation of primary sclerosing cholangitis (PSC) to systemic lupus erythematosus (SLE) causality plot. **A** Scatter plot of PSC-related single-nucleotide polymorphisms (SNPs) and their associated risk for SLE. **B** Forest plot of PSC-related SNPs and their associated risk for SLE. **C** Leave-one-out” plots for the causal association between genetically predicted PSC and SLE

Sixteen PSC-related SNPs were identified and are listed in Supplementary Table 1, with the impact of each SNP on SLE illustrated in Fig. 2A and B. The genes corresponding to these SNPs are *SH2B3, CLEC16A, TTC34, FOXP1*, *SGSM1*, *KIAA1109*, *RNF123*, and *CYP21A1P*.

Among them, *SH2B3, CLEC16A,* and *TTC34* are mainly involved in immune regulation and autoimmune diseases, while other genes are involved in regulating neurological, adrenal gland, and skeletal muscle-related diseases. Five methods were used to evaluate the effects of PSC on SLE. The IVW and weighted median results (Table 2) revealed that PSC may be associated with an increased risk for SLE; however, the results of the other three methods did not support this finding. IVW and MR-Egger results revealed heterogeneity in these IVs (*P* < 0.05, Table 3). The leave-one-out analysis (Fig. 2C) revealed that removing any single SNP had no significant effect on the results. The MR-Egger regression results revealed no significant level of pleiotropy (Table 3).

**Table 2:** Causal relationship between PSC and SLE

Exposure	Outcome	SNPs, n	Statistical method	OR	95% CI		*P*
					Lower	Upper	
PSC	SLE	16	MR egger	1.29	0.96	1.71	0.109
			Weighted median	1.1	1.01	1.2	0.027
			IVW	1.33	1.1	1.61	0.004
			Simple mode	1.13	1	1.29	0.067
			Weighted mode	1.05	0.98	1.12	0.176
SLE	PSC	27	MR egger	1	0.85	1.18	0.982
			Weighted median	1.06	0.99	1.13	0.104
			IVW	1.07	0.99	1.16	0.072
			Simple mode	1	0.91	1.11	0.937
			Weighted mode	1.04	0.96	1.12	0.346

CI:  confidence interval; OR:  odds ratio; PSC:  primary sclerosing cholangitis; SLE:  systemic lupus erythematosus; SNP:  single-nucleotide polymorphism

**Table 3:** Sensitivity and polymorphism analysis results of MR

Exposure-outcome	IVW (heterogeneity)	MR egger (heterogeneity)	MR egger (pleiotropy)	
	*P* value	*P* value	*P* value	Intercept
PSC-SLE	1.33E-54	6.80E-55	0.773	0.019
SLE-PSC	1.84E-06	2.42E-06	0.366	0.028

PSC:  primary sclerosing cholangitis; SLE:  systemic lupus erythematosus

### Impact of SLE on PSC

**Fig. 3:**
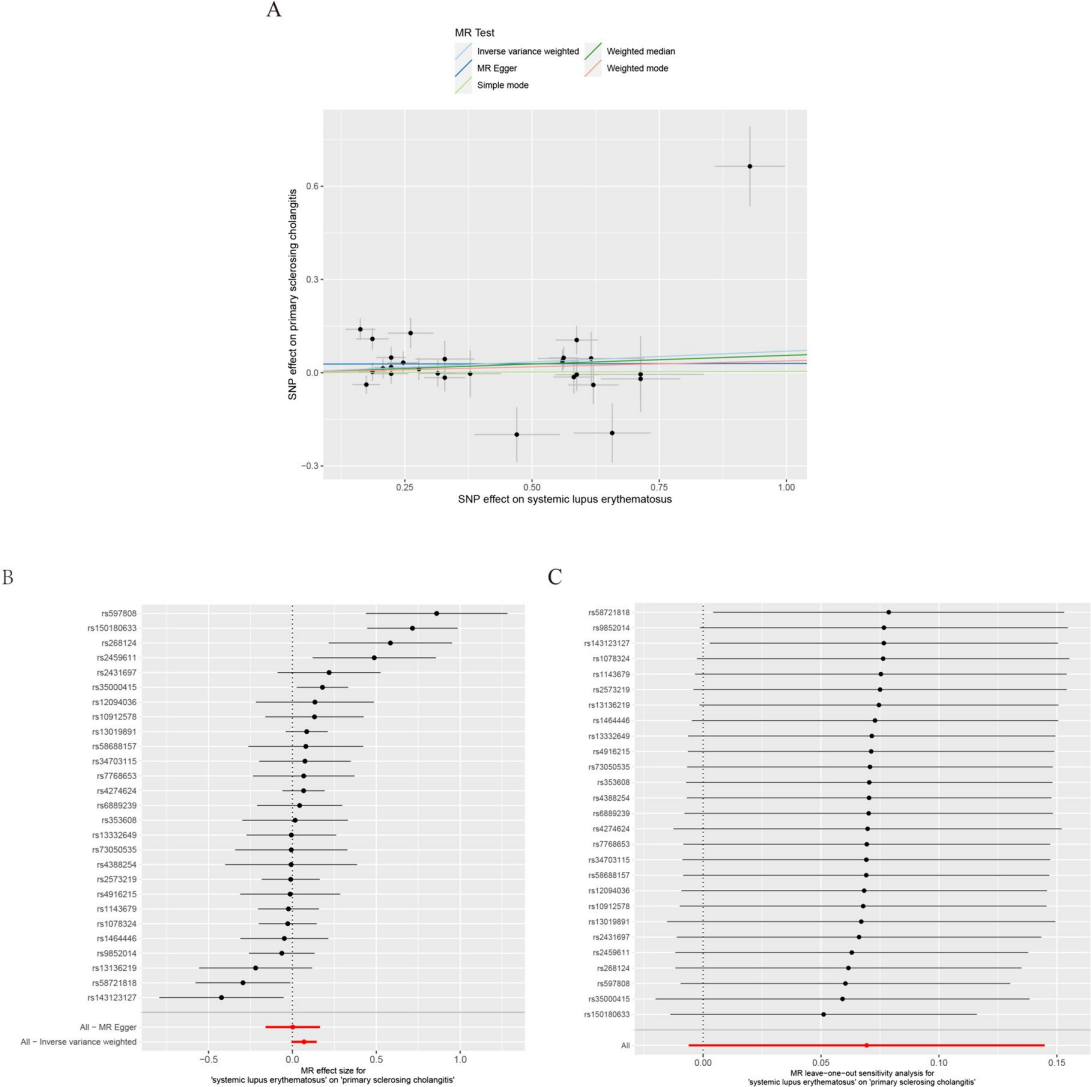
Mendelian randomization estimation of systemic lupus erythematosus (SLE) to primary sclerosing cholangitis (PSC) causality plot. **A** Scatter plot of SLE-related single-nucleotide polymorphisms (SNPs) and their associated risk for SLE. **B** Forest plot of SLE-related SNPs and their risk to SLE. **C** “Leave-one-out” plots for the causal association between genetically predicted SLE and PSC

After removing 4 SNPs related to IBD, 29 SNPs related to SLE were obtained. The impact of each SNP on PSC (Supplementary Table 2) is shown in Fig. 3A and B. Results of the five analysis methods (Table 2) revealed no correlation between SLE and PSC (P > 0.05). The IVW and MR-Egger results revealed heterogeneity in these instrumental variables (P < 0.05, Table 2). MR-Egger regression results revealed no significant level of pleiotropy. The leave-one-out analysis (Fig. 3C) revealed that removing any single SNP had no significant effect on the results. The MR-Egger regression results revealed no significant level of pleiotropy (Table 3).

## Discussion

To the best of our knowledge, this was the first study to use bidirectional MR analysis and large-scale GWAS data to demonstrate a relationship between PSC and SLE. Our results revealed that PSC, as a positive factor, is of great significance in promoting the occurrence of SLE. However, our study showed no evidence supporting the impact of SLE on PSC.

Studies have shown that PSC is linked to a variety of autoimmune diseases such as type I diabetes, thyroid disease, and rheumatoid arthritis [21]. Some studies have found that patients with IBD exhibit an increased risk for developing autoimmune disease(s) [22, 23], and two-thirds of individuals with PSC often have IBD [24]. Because patients with PSC exhibit a high incidence of IBD, it is exceedingly difficult to determine whether the increased probability of autoimmune diseases in PSC is due to IBD or whether PSC itself can cause development of autoimmune disease(s). Saarinen et al. [6] compared the incidence of autoimmune diseases in patients with PSC and IBD without liver disease and found that those with PSC exhibited a higher incidence of autoimmune diseases than IBD patients without liver disease.

SLE is an autoimmune disease that affects most organs throughout the body, including the liver. It has been reported in the literature [25] that individuals with SLE have a 25–50% probability of developing abnormal liver function during their lifetime. Drug-induced liver injury is the most common cause of abnormal liver function.

Autoimmune diseases are a constellation of conditions caused by intolerance of the autoimmune system to self-antigens and their immune response to self-tissues. The causative factors of autoimmune diseases are similar and multiple autoimmune diseases are commonly observed in a single individual. With the emergence of GWAS, an increasing number of studies have confirmed that multiple gene loci are associated with ≥ 1 autoimmune disease(s) [26]. For example, the protein tyrosine phosphatase non-receptor type 22 *(PTPN22*, rs2476601) have been found to be associated with type 1 diabetes [27], autoimmune thyroid disease [28], SLE [29], and rheumatoid arthritis [30]. *NOTCH4* is also associated with alopecia areata [31] and rheumatoid arthritis [32]. A study by Cotsapas et al. [33] investigating the association between 107 immune-mediated disease SNPs and autoimmune diseases found that nearly one-half of these SNPs were associated with multiple immune-mediated diseases.

Although patients with PSC and concurrent SLE are rare, there are some reports [9] describing the coexistence of these two diseases, suggesting that a common pathogenic mechanism may exist. There are many autoantibodies involved in autoimmune diseases, and some antibodies are of great significance in disease diagnosis and treatment. As an autoimmune disease that contains many autoantibodies. Anti-dsDNA and anti-smooth muscle autoantibodies are of great significance in the diagnosis of SLE. They also contain various autoantibodies [5]. For example, anti-bactericidal/permeability increasing protein antibodies are present in 5%–46% of patients, and anti-lactoferrin antibodies are present in 4–54% of patients with PSC; these antibodies can also be detected in patients with SLE [34, 35]. In addition, the study by Granito et al. [36] demonstrated that 30% of patients with PSC were antinuclear antibody (ANA)-positive, whereas 93% of SLE patients were ANA-positive. Although many autoantibodies have been detected in patients with PSC [5], however, their specificity are usually low and their significance remains unclear. Studies [37] have found that autoantibodies associated with primary biliary cholangitis, one of the autoimmune liver diseases, are common in SLE, even in the absence of elevated liver enzymes. Similarly, autoantibodies co-expressed in patients with SLE and PSC may also be found in SLE patients without clinical symptoms of PSC patients. This may limit the diagnostic significance of these autoantibodies in PSC. Further relevant research is still needed in the future to improve the accuracy of diagnosis of PSC.

Currently, there are few studies investigating the correlation between PSC and SLE, and systematic retrospective research investigating the relationship between these two diseases is lacking. Because observational studies are prone to confounding factors and reverse causation, even if there is a statistically significant result, the exact cause of the disease cannot be determined. Randomized controlled trials (RCTs) are considered good alternatives to observational studies, and have been unanimously considered to provide strong support for studying the causal factors of diseases. However, RCTs have certain limitations [38]. RCTs usually require considerable time and financial support, and the complexity of the research design and ethical aspects of the subject have restricted their development. Currently, MR has become a new epidemiological method for studying diseases. Based on whole-genome sequencing data, MR studies can use genetic variation as an IVs to investigate the relationship between exposure factors and outcomes. MR studies can partially resolve confounding and reverse causation, and provide stronger support for disease causation.

The strength of this study is that we used the largest genetic variation data for the two diseases in the GWAS data and the MR method to bidirectionally evaluate the association between PSC and SLE. In addition, we implemented strict criteria to screen IVs and remove IVs that may lead to polymorphisms, and used five MR methods to confirm our findings.

However, the current study had some limitations. First, although we implemented strict criteria to screen for IVs, the limited nature of MR studies may have led to potential bias. Second, regarding data from the MR study, SLE was a European population, whereas PSC was mainly a European population, which limits the generalizability of the results to other populations. Third, no positive results for SLE in PSC were found, and further confirmation may be needed in a larger population. Fourth, PSC mainly occurs in males, whereas SLE mainly occurs in females. No sex- or age-specific GWAS data were available.

## Conclusion

In conclusion, we found that PSC was an independent risk factor for SLE through MR analysis; however, further studies are needed to elucidate the biological mechanism underlying the association between the two diseases.

### Supplementary Information

Supplementary Material 1.

Supplementary Material 2.

## Data Availability

The data obtained in this article can be downloaded from the GWAS database.

## Abbreviations

ANA: Antinuclear antibody
CI: Confidence interval
GWAS: Genome-wide association studies
IBD: Inflammatory bowel disease
IVs: Instrumental variables
IVW: Inverse variance weighting
MR: Mendelian randomization
OR: Odds ratio
PSC: Primary sclerosing cholangitis
RCTs: Randomized controlled trials
SNPs: Single-nucleotide polymorphisms
SLE: Systemic lupus erythematosus
