# Supplementary material for: Causal relationship between primary sclerosing cholangitis and systemic lupus erythematosus: a bidirectional Mendelian randomization study
Source: Eur J Med Res. 2024 Jun 28;29:351. doi: 10.1186/s40001-024-01941-1 (PMC11212221; doi:10.1186/s40001-024-01941-1)
Supplement: Supplementary file 1 — Supplementary Material 1. [file 40001_2024_1941_MOESM1_ESM.docx]

| **Supplementary Table 1.** Sixteen PSC-related SNPs. | | | | | | | |
| --- | --- | --- | --- | --- | --- | --- | --- |
| rs3184504 | rs3131781 | rs725613 | rs72837826 | rs231389 | rs10909839 | rs80060485 | rs114581973 |
| rs41316239 | rs145832854 | rs4817988 | rs13119723 | rs4147359 | rs9858213 | rs139010734 | rs34645399 |

Abbreviations: PSC = primary sclerosing cholangitis
