# Supplementary material for: Causal relationship between primary sclerosing cholangitis and systemic lupus erythematosus: a bidirectional Mendelian randomization study
Source: Eur J Med Res. 2024 Jun 28;29:351. doi: 10.1186/s40001-024-01941-1 (PMC11212221; doi:10.1186/s40001-024-01941-1)
Supplement: Supplementary file 2 — Supplementary Material 2. [file 40001_2024_1941_MOESM2_ESM.docx]

| **Supplementary Table 2.** 27 SLE-related SNPs. | | | | | | | | |  |
| --- | --- | --- | --- | --- | --- | --- | --- | --- | --- |
| rs1078324 | rs12094036 | rs13332649 | rs150180633 | rs2573219 | rs35000415 | rs4388254 | rs58721818 | rs73050535 | |
| rs10912578 | rs13019891 | rs143123127 | rs2431697 | rs268124 | rs353608 | rs4916215 | rs597808 | rs7768653 | |
| rs1143679 | rs13136219 | rs1464446 | rs2459611 | rs34703115 | rs4274624 | rs58688157 | rs6889239 | rs9852014 | |
